# Foot-and-mouth disease: genomic and proteomic structure, antigenic sites, serotype relationships, immune evasion, recent vaccine development strategies, and future perspectives

**DOI:** 10.1186/s13567-025-01485-0

**Published:** 2025-04-07

**Authors:** Alyaa Elrashedy, Mohamed Nayel, Akram Salama, Ahmed Zaghawa, Rehan M. El-Shabasy, Mohamed E. Hasan

**Affiliations:** 1 Department of Animal Medicine and Infectious Diseases (Infectious Diseases), Faculty of Veterinary Medicine, University of Sadat City, Sadat City, Egypt; 2 Faculty of Health Science Technology, Borg Al Arab Technological University (BATU), Alexandria, Egypt; 3 Chemistry Department, The American University in Cairo, AUC Avenue, New Cairo, 11835 Egypt; 4 Department of Chemistry, Faculty of Science, Menoufia University, Shebin El-Kom, 32512 Egypt; 5 Bioinformatics Department, Genetic Engineering and Biotechnology Research Institute, University of Sadat City, Sadat City, Egypt

**Keywords:** Foot-and-mouth disease, immune evasion, antigenic sites, matching, vaccines, peptide vaccines

## Abstract

Foot-and-mouth disease (FMD) is a highly contagious and transmissible disease that can have significant economic and trade repercussions during outbreaks. In Egypt, despite efforts to mitigate FMD through mandatory immunization, the disease continues to pose a threat due to the high genetic variability and quasi-species nature of the FMD virus (FMDV). Vaccines have been crucial in preventing and managing FMD, and ongoing research focusses on developing next-generation vaccines that could provide universal protection against all FMDV serotypes. This review thoroughly examines the genetic structure of FMDV, including its polyprotein cleavage process and the roles of its structural and non-structural proteins in immune evasion. Additionally, it explores topics such as antigenic sites, specific mutations, and serotype relationships from Egypt and Ethiopia, as well as the structural changes in FMDV serotypes for vaccine development. The review also addresses the challenges associated with creating effective vaccines for controlling FMD, particularly focusing on the epitope-based vaccine. Overall, this review offers valuable insights for researchers seeking to develop effective strategies and vaccines for controlling FMD.

## Introduction

Foot-and-mouth disease (FMD) is a contagious disease that significantly impacts the economy and trade regarding both domestic and wild cloven-hoofed animals. In areas where FMD is endemic, the total economic loss is estimated to range from $6.5 to $21 billion each year. Even countries that are free from FMD still incur costs exceeding $1.5 billion [1]. The situation is especially challenging in Africa, where the disease leads to over $2 billion in losses each year [2].

The causative agent of FMD is the foot-and-mouth disease virus (FMDV), which belongs to the *Picornaviridae* family and the *Aphthovirus* genus [3]. The disease spreads primarily through direct or indirect contact with infected animals, their secretions, or contaminated feed. It can also be transmitted over large distances via airborne infected aerosols [4].

Clinical signs of FMD include fever and severe vesicular sores on the extremities, oral cavity, udder, teats, and nasal area. Additional symptoms may include lameness, reduced milk production, and a decline in overall health [5]. Notably, adult animals tend to have lower fatality rates compared to calves, which often succumb to myocarditis, resulting in higher morbidity and mortality rates among younger animals [6].

Immunologically, FMDV comprises seven distinct serotypes: A, O, C, Asia-1, and the Southern African Territories (SAT1-3), with over 65 genetic lineages and topotypes [7]. In Egypt, the serotypes identified as the causative agents are A, O, and SAT2. The ongoing introduction of new lineages from external sources creates an unstable environment that complicates the selection of suitable vaccination antigens [8].

The first documented outbreak of FMD in Egypt was caused by serotype SAT2 in 1950 [9]. In 2012, SAT2 re-emerged and led to a significant increase in mortality rates among ruminants [10]. There are persistent concerns regarding historical outbreaks, particularly those involving serotypes A and O in 1958 and subsequent years [11].

In February 2006, serotype A was responsible for 12 outbreaks across seven Egyptian governorates. These included one outbreak in Behera, two in Alexandria, one in Cairo, five in Dumyat, one in Dakahlia, one in Menoufia, one in Fayum, and six in Ismailia [12]. Additionally, there is a history of epidemics caused by serotype O in Egypt [13]. Despite mandatory vaccination, FMD outbreaks continue nationwide, with the novel serotype A topotype Africa G-IV strain emerging in 2012, followed by sporadic reports of outbreaks in 2016 and 2018 [14].

In 2019, a new sublineage of SAT2 was discovered, which resulted in severe clinical symptoms in buffaloes [15]. A study conducted on outbreaks from 2018 to 2022 in two provinces, Kafr El-Sheikh and Beheira, revealed that the dominant serotypes in 2018 were A and SAT2, while in 2022, only serotype A was identified [16].

Epidemiological and genetic studies conducted in Egypt from 2022 to 2024 indicated that the endemic persistence of serotype O is associated with the East Africa 3 (EA-3) topotype, while serotype A corresponds to the Africa G-IV topotype [17]. Consequently, researchers performed a risk analysis and concluded that the use of appropriate vaccines could aid in the prevention and control of FMD in Egypt [18].

Mass vaccination is recognized as the primary method for managing FMD. However, the current commercially available inactivated vaccines present significant challenges due to their limited cross-protective efficacy, not only among the seven distinct serotypes of the virus but also within individual serotypes. This situation is further complicated by the considerable genetic variability that arises during RNA replication, along with frequent instances of inter- and intra-serotype recombination [19].

This comprehensive overview provides important insights into the biological structure of FMDV, including its complex interactions with the immune response, the relationships between different FMDV serotypes, and the antigenic sites they target. It also examines the various strategies being developed for FMD vaccine production.

## Deconstructing FMDV for vaccine design

A better understanding of the biology of FMDV has facilitated the design and development of innovative potential vaccines [20]. FMDV is a small, non-enveloped icosahedral capsid composed of 60 copies of four structural proteins: VP1, VP2, VP3, and VP4. It consists of a linear, single-stranded RNA (ssRNA) genome that is non-segmented, has a positive sense, and spans approximately 8.5 kb nucleotides [3].

The RNA genome of FMDV contains a single large open reading frame (ORF) that functions as messenger RNA (mRNA) and encodes a polyprotein of around 7000 nucleotides. This ORF is flanked by an extended 5′-untranslated region (UTR) of approximately 1,300 nucleotides and a shorter 3′-UTR of around 90 nucleotides, followed by a poly(A) tail [21, 22]. The polyprotein is cleaved by viral-encoded proteases (L and 3C), resulting in the separation of the polyprotein into three segments: P1, P2, and P3 portions. This process yields a total of 15 mature virus proteins.

**Figure 1:**
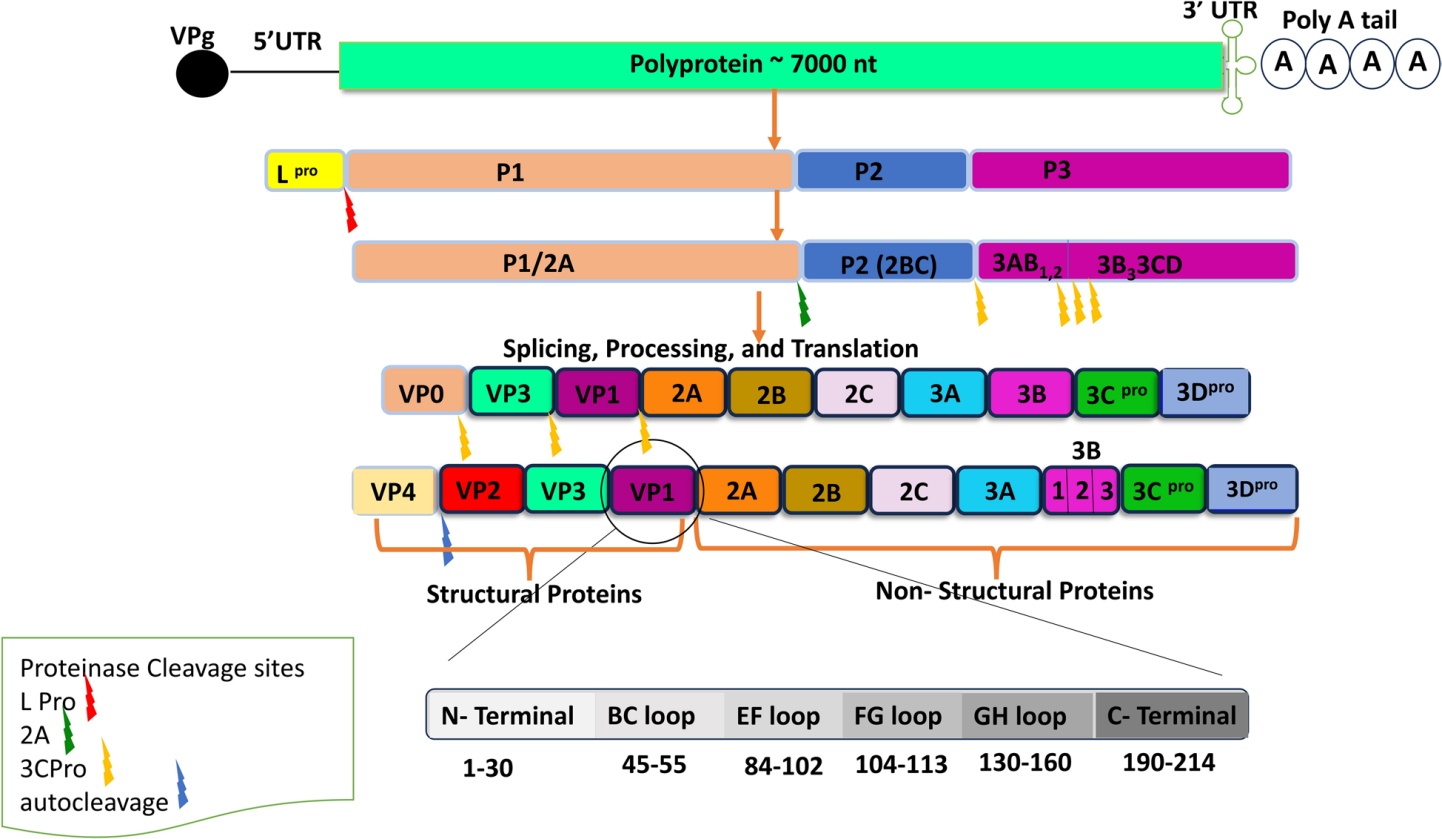
**FMDV genome structure, protein cleavage, and VP1 antigenic composition**.

The P1 segment encodes the leader proteinase (L^pro^) and the structural proteins VP1 (1D), VP2 (1B), VP3 (1C), and VP4 (1A). The P2 segment encodes the non-structural proteins 2A, 2B, and 2C, while the P3 segment encrypts 3A, 3B, the viral protein genome (VPg) (1B, 2B, and 3B), 3C protease, and 3D polymerase. The non-structural proteins (NSPs) primarily play roles in protein synthesis, RNA replication, and immune evasion (Figure 1) [6].

Picornaviruses lack a proofreading mechanism, which allows for the emergence of quasi-species. These are a dynamic population of viruses that continuously mutate, evolve, and adapt to their environments, demonstrating resilience against immune responses and antiviral treatments [23]. The consensus sequence of FMDV alterations occurs slowly and gradually, with approximately 0.5 to 1.0% of the genome altered per year, translating to around 1–2 nucleotide changes per week. This genetic variability aids in tracking the spread of the virus during outbreaks [24].

Among the serotypes, serotype O is the most widespread, although it is not the most antigenically diverse; serotype A exhibits a higher level of diversity. The amino acid homology between these two serotypes is estimated to be around 86%. The viral proteins VP1, VP2, and VP3 are located on the surface of the virus, forming an eight-stranded β-barrel structure, while VP4 is located internally with minimal secondary structure [25].

VP1 is the most variable gene, displaying a 30% to 50% difference between serotypes, which is crucial for serotype identification [26]. Key antigenic sites are located within different loops of the structural viral proteins, including the N-terminal, B-C loop, E-F loop, F-G loop, G-H loop, and the C-terminal. Notably, the VP1 protein contains highly conserved RGD (arginine-glycine-aspartic acid) motifs in the G-H loop, which are essential for the virus to enter cells by binding to host integrin receptors. These receptors consist of a diverse family of 24 α–β heterodimers that recognize the RGD sequence in their natural ligands [27].

Furthermore, the G-H loop features a hypervariable region (HV) that affects immune evasion and antibody binding. Intact virus particles, especially those with the G-H loop of VP1, are significantly immunogenic [28]. Research by Fernandez-Sainz et al. showed that substituting the hypervariable epitope of the G-H loop of VP1 with a xenoepitope from the porcine reproductive and respiratory syndrome (PRRS) virus on an adenovirus-vectored FMDV vaccine (Ad5-FMD) led to reduced immunogenicity and efficacy in swine, highlighting the original epitope’s critical role in generating protective immunity [29].

Additionally, variability in vaccine response can be attributed to factors such as antigen choice, dose, and environmental influences. A meta-analysis on the polypeptide vaccine (PPV) assessed its efficacy and effectiveness, revealing a dose-dependent protective effect in pigs. A dose of PPV ≤ 1 mg protected 69.41% of pigs, which is lower than the WHO standard of 75%. However, a PPV dose of ≥ 2 mg protected 97.22% of pigs [30].

### Leader protein (L^Pro^)

The Leader (L) protein, found at the N-terminal end of the viral polyprotein, exists in two forms: Lb and Lab. These forms arise from the initial step of protein synthesis at specific sites. This dual-initiation site is universally conserved across all FMDV serotypes and strains, although the evolutionary advantage of this feature remains unclear.

The L protein serves two crucial functions. First, it acts as a papain-like cysteine protease, facilitating cleavage at the L/P1 region of the polyprotein. This cleavage also activates the termination of translation initiation factors, resulting in the cessation of cap-dependent protein synthesis [31]. Additionally, the L protein plays other roles that contribute to viral replication and inhibit host defense mechanisms.

Notably, the removal of the Lb coding sequence significantly weakens the virus’s infectivity, suggesting that developing strains with reduced virulence could be beneficial for classical vaccine production [32].

### The capsid precursor P1-2A

The L^pro^ enzyme cleaves the P1-2A precursor at the N-terminal region, leading to the acylation of the exposed residue, which is essential for effective virus replication. The cleaved P1-2A precursor is then split via 3C protease (3C^Pro^) into VP0, VP3, VP1, and a short 2A peptide (Figure 1). During capsid assembly, the cleavage of VP0 into VP4 and VP2 is facilitated by the cellular chaperone HSP90 and the myristoylation of the N-terminus of P1-2A. This step is crucial for the production of virus capsid pentamers [33]. Additionally, a conserved motif within VP1, referred to as YCPRP, is important for the processing of the capsid precursor 3C^Pro^. Alterations in this motif can lead to misfolding and accelerated degradation of the capsid precursor [34]. In summary, the P1-2A region in FMDV plays a vital role in polyprotein processing, producing the structural proteins necessary for generating infectious virions and empty capsids.

### The 2BC precursor

The 3C^pro^ enzyme cleaves the 2BC precursor region to produce two non-structural proteins, 2B and 2C. The 2B NSP is a short hydrophobic protein made up of 154 amino acids and is reported to have viroporin functions. In contrast, the 2BC protein has an inhibitory effect on protein trafficking to the cell surface, which may reduce exposure to the host immune system. This functional characteristic requires the simultaneous expression of both the 2B and 2C proteins.

### The 3ABCD precursor

The cleavage of the 3ABCD precursor is a crucial step in the complex viral polyprotein processing pathway. This process leads to the formation of the 3A peptide, three distinct versions of the 3B protein (3B1, 3B2, and 3B3), as well as the proteins 3C^pro^ and 3D^pol^ [33] (Figure 1). The exact function of the FMDV 3A protein (153 amino acids) remains unclear; however, it may influence host specificity and virulence through amino acid substitutions. Its elongated C-terminal domain distinguishes it from most other picornaviruses. Unlike the 3A non-structural protein of other picornaviruses, which plays a significant role in viral replication, the replication of FMDV replication does not depend on the same cellular factors.

The vaccine, which is free of oil emulsion and includes A-HSP70 and A -3A strains, stimulated immune responses during the early, middle, and long-term stages in both mice and pigs. This modification features the surface display of T-cell epitopes from HSP-70 and 3A-NSP, enhancing the initial cellular immune response following vaccination and promoting the development of lasting memory responses [35].

A unique characteristic of FMDV is that the three copies of the 3B protein, which are intricately linked to the 5-prime end of ± ssRNA, act as essential primers for initiating viral replication [36]. The 3C^pro^ protein is responsible for cleaving protein junctions and is considered a potential target for antiviral agents [33] (Figure 1). Similarly, 3D^pol^ has gained attention as a target for antiviral development [36]. The entire viral replication cycle occurs within the cellular cytoplasm, and advancements in virology have facilitated the design and construction of systems capable of expressing cleaved capsid proteins and assembling them into virus-like particles (VLPs).

## Structural and non-structural proteins of FMDV and their strategies for immune evasion

The antiviral immune response is triggered by the innate immune signaling pathway, which leads to the production of immune molecules such as interferon-type I (INF), interferon-stimulated genes (ISGs), and inflammatory cytokines in reaction to various viruses. Pattern recognition receptors (PRRs), including toll-like receptors (TLRs), retinoic acid-inducible gene-I (RIG-I)-like receptors (RLRs), and nucleotide-binding and oligomerization domain (NOD)-like receptors (NLRs), play a crucial role in identifying pathogen-associated molecular patterns (PAMPs). Among the RLRs family, melanoma differentiation-associated gene 5 (MDA5) and RIG-I are significant members that detect cytosolic viral RNAs, especially from picornaviruses, making them prime targets for immune evasion by viruses [37].

The VP1 and VP3 proteins of FMDV primarily inhibit the generation of INF and dampen the antiviral response in host organisms. In contrast, the other two proteins, VP2 and VP4, facilitate the replication of the virus through processes such as autophagocytosis, among others. Understanding these structural proteins is crucial for the design and development of vaccines [38].

VP1 plays a critical role in various processes, including the induction of neutralizing antibodies (nAbs), the mediation of humoral and cellular immune responses, the initiation of cell death in the host, and the facilitation of viral replication. It has been observed that VP1 interacts with sorcin, a soluble calcium-binding protein related to resistance, which helps to impede the production of IFN-type I and disrupt the nuclear factor-kappa B (NF-κB) signaling pathway, leading to sustained infection [39].

Moreover, the interaction between VP1 and DNAJA3 reduces VP1’s inhibitory effect on the IFN-β signal transduction pathway, thereby restraining virus replication [40]. Simultaneously, VP1 mitigates the inhibitory effect of ribosomal protein SA (RPSA) on the mitogen-activated protein kinase pathway (MAPK), thus promoting viral replication [41].

The VP2 protein also reduces the IFN-type I pathway by interacting with the host’s heat shock protein member 1 in the B family (HSPB1), which enhances viral replication [42]. Although VP3 is more conserved than VP1, it plays a crucial role in viral assembly and the suppression of the innate immune response. Research has shown that the arginine residue 56 in VP3 is associated with FMDV virulence.

The FMDV VP3 protein also facilitates the translocation of tumor protein 53 (TP53) from the nucleus to the mitochondria, leading to apoptosis and autophagy—both of which are essential for FMDV replication. The Gly129 residue is conserved among various picornaviruses, underscoring its significance in developing broad-spectrum antivirals and genetically engineered vaccines against these viruses. Gly129 is critical for VP3’s interaction with TP53 and for promoting autophagy. When Gly129 is substituted with Ala129, VP3 loses its ability to regulate autophagy, resulting in a significant decrease in viral replication and pathogenicity. This indicates that VP3-induced autophagy is essential for the virus's proliferation [43].

Additionally, VP3 interacts with Janus kinase 1 (JAK1), leading to its degradation and the subsequent inhibition of the IFN-γ signaling pathway. This suppression blocks the phosphorylation, dimerization, and nuclear accumulation of the signal transducer and activator of transcription 1 (STAT1), which are crucial for the antiviral response. As a result, the IFN-type II signal pathway is inhibited, facilitating the virus’s evasion of the innate immune system [44].

Furthermore, the C-terminal region of the VP3 protein (111–220 aa) can interact with a virus-induced signaling adapter (VISA) and inhibit its expression, ultimately suppressing INF-β production [45]. Additionally, TANK-binding kinase-1 (TBK-1) and microRNA-1307 work together to degrade VP3, enhancing the host’s immune response [46].

The VP0 protein plays a role in suppressing the innate immune response by promoting the degradation of VISA through an apoptotic pathway [47]. Similarly, VP4 disrupts the antiviral innate immunity that is activated by p53, which normally enhances the expression of ISGs and Nucleoside diphosphate kinase 1 (NME1). This disruption is facilitated by specific regions of the VP4 protein, specifically the 15–30 and 75–85 residues [48]. In summary, FMDV employs various strategies, driven by different structural proteins, to evade the host's natural immune response. This enables the virus to enhance its reproduction and develop techniques for immune evasion.

On the opposite side of the NSPs, L^pro^ plays a key role in facilitating FMDV replication while also inhibiting the host immune response through various mechanisms. One of these mechanisms involves cleaving eukaryotic initiation factor (eIF4G), which prevents the translation of host proteins. L^pro^ also inhibits the levels of IFN regulatory factor 3/7 (IRF3/7), thereby restraining the transcription of IFN-β mRNA [49]. Furthermore, it prevents the ubiquitination of the signal of native immunity molecules such as TNF receptor-associated factor 3/6 (TRAF3/6), TBK-1, and RIG-I.

**Figure 2:**
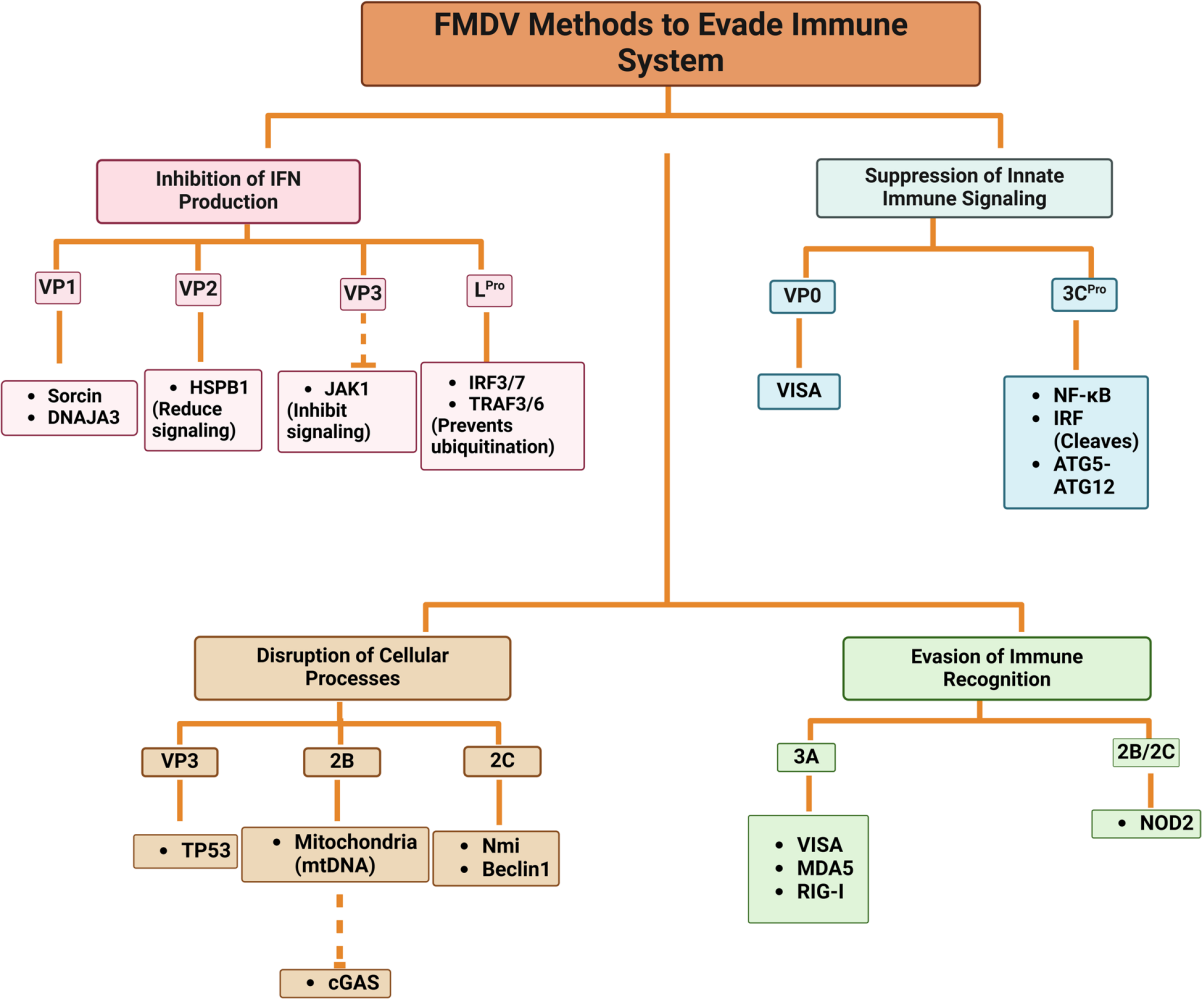
**FMDV immune evasion strategies targeting host immune system.** Key structural and non-structural proteins of FMDV, including VP1, VP2, VP3, L^pro^, 2B, 2C, 3A, and 3C^Pro^ target various components of the host immune system. These proteins inhibit interferon (IFN) production, suppress innate immune signaling pathways, disrupt critical cellular processes, and evade immune recognition. These all facilitate viral replication and persistence in the host.

During the early stages of FMDV infection, L^pro^ interacts with transcription factors to block the expression of ISGs as well as IFNs [50]. Additionally, L^pro^ inhibits the formation of SGs by cleaving SG scaffold proteins G3BP1 and G3BP2, which could significantly contribute to the suppression of the IFN type I cascade [51] (Figure 2).

In the case of the FMDV 2B protein, it disrupts the host’s secretory pathway, inhibiting IFN responses by suppressing the expression of MDA5 and RIG-I through its C-terminal amino acids (126–154) [52]. This disruption involves ion transport, increases the permeability of the host cell membrane, and disturbs Ca^2+^ equilibrium, potentially aiding in viral replication. If this function is impaired, it poses a life-threatening challenge for the virus [53].

Additionally, NOD2 plays a critical role in the immune system’s recognition processes by triggering IFN-β production and activating NF-ĸB. A study by Liu et al. demonstrated that NOD2 can slow down the replication of FMDV. However, the NSPs 2B, 2C, and 3Cpro of FMDV can evade the immune response by reducing the levels of NOD2 [54].

During FMDV replication, the 2B protein damages mitochondria, leading to the release of mitochondrial DNA (mtDNA). This released mtDNA activates cyclic GMP-AMP synthase (cGAS), which triggers an antiviral response by regulating IFN-β production. cGAS is crucial for controlling FMDV replication; if it does not function properly, it results in higher mortality in infected mice. Furthermore, the FMDV 2C protein interferes with the cGAS-stimulator of the interferon genes (STING) pathway, with specific amino acids in 2C playing a key role in this interference [55]. The 2C protein also activates NLRP3 inflammasomes, promoting the production of IL-1β [56].

Studies suggest that targeting 2B could lead to potential strategies for developing vaccines against FMDV and attenuating the virus.

The FMDV 3A protein employs complex mechanisms to evade the host immune response. It downregulates the expression of proteins such as VISA, MDA5, and RIG-I, and inhibits the phosphorylation of IRF3 through pathways that depend on the DEAD-box family protein (DDX56) [57]. Additionally, it upregulates leucine-rich repeat-containing protein 25 (LRRC25), which suppresses G3BP1-mediated RLH signaling [58].

The FMDV 3C^pro^ plays a crucial role in the transcriptomics and proteomics of host cells. It achieves this by cleaving essential factors and regulators necessary for the function of cellular DNA-dependent RNA polymerases I, II, and III. Notable proteins that 3C^pro^ targets include the transcription activator p53, TATA-box binding protein, cyclic AMP-responsive element binding protein, octamer-binding protein, and DNA polymerase III [59].

Furthermore, 3C^pro^ inhibits host protein synthesis by cleaving eIF4G and eIF4A. It also cleaves the NF-κB essential modulator (NEMO) but at the glutamine (Q 383) site, which interferes with NF-κB and IRF activity [60]. By targeting the ATG5-ATG12 complex, 3C^pro^ disrupts autophagy processes [61]. Additionally, it hampers JAK-STAT signaling through karyopherin α1 (KPNA1), as shown in the study referenced here [62].

Moreover, the 3C^pro^ protein is involved in regulating its life cycle by binding to the FMDV internal ribosome entry site (IRES) and enhancing replication through its interaction with the src-associated protein in mitosis (Sam68) [63].

The specific role of 2C in genome is not yet fully understood, but it contains helicase motifs and has demonstrated the ability to form hexameric structures. Additionally, it interfaces with the host protein Nmi (N-myc and STAT interactor), which suggests that it may play a role in FMDV-induced apoptosis [64]. Furthermore, the inactivation of Beclin1 by 2C hinders the fusion of FMDV-containing autophagosomes with lysosomes, thereby preventing the degradation of the virus [65]. Moreover, 3D^pol^ interacts with DEAD-box RNA helicase 1 (DDX1) to inhibit FMDV replication and increase the production of IFN-β, which enhances the innate antiviral immune response [66]. These complex mechanisms collectively facilitate FMDV replication and enable the virus to evade the immune system.

The various structural and non-structural proteins of FMDV play critical roles in inhibiting innate immune pathways. They achieve this through various interactions and cleavage events that disrupt essential antiviral defenses. Although significant insights have been gained, there are still gaps in our understanding of the specific mechanisms involved, particularly with proteins like 2C and its precise roles in viral genome replication. These findings highlight the ongoing need for further research to fully clarify these mechanisms, which could lead to innovative approaches for vaccine development and therapeutic strategies against FMDV.

## FMDV serotypes relation and matching

The rapid evolution of FMDV, accelerated by high mutation rates and significant antigenic diversity, often results in discrepancies between circulating strains and existing vaccines. The antigenic mismatch is a major reason for vaccination failures, underscoring the need for continuous surveillance to identify new strains and revise vaccine formulations accordingly. Addressing this challenge requires a proactive approach to vaccine production, focusing on cross-protective immunity and possibly integrating computational methods to predict antigenic drift and improve vaccine strain selection [67].

It is important to note that infection with one serotype of FMDV does not provide cross-protection against other serotypes. Additionally, animals that recover from FMDV infection may become carriers of the virus while remaining susceptible to other serotypes. The application of computational methods to analyze antigen–antibody (Ag-Ab) interactions and forecast cross-antigenicity is essential for developing modified vaccines that elicit strong, broad, and lasting immune responses against various FMDV strains [68].

The findings from Egypt in 2017 and 2018 indicated that serotype O was linked to the EA-3 topotype, displaying a 12.7% difference from the O-IRN-8–2005-Pan-Asia-2 vaccinal strain. In 2018, serotype SAT2 was associated with the VII topotype but it was closely related to strains collected from Libya in 2012, sharing a 94.3% amino acid identity. This similarity distinguished it from the SAT2 strains that have been circulating in Egypt since 2012, providing evidence for the emergence of a new topotype within serotype SAT2 [69].

In another study, sequencing and phylogenetic analysis of the VP1 protein from serotypes O, A, and SAT2 showed that the O serotype exhibited a close genetic relationship with O/SUD/8/2008, sharing 94.6% identity. However, it also displayed a notable 14.6% difference from the vaccinal strain (O/PanAsia-2/ topotype ME-SA). Additionally, serotypes A and SAT2 showed a close resemblance to current Egyptian field and vaccine strains, specifically A/EGY/1/2012 (topotype Asia-1, lineage Iran-05) and the vaccine strain SAT2/ALX-12 (topotype VII), with 96.4% and 95.3% shared identity, respectively [13].

Moreover, in 2020, sequence analysis of VP1 from FMDV samples in Port-Said Governorate revealed that the field strains were classified as serotype A-Africa topotype, Genotype IV. These strains displayed close genetic affinity to isolates from the Animal Health Research Institute (AHRI) in 2020, with nucleotide similarity ranging from 97.1% to 99.74%. There was a genetic variation of 5.63–7.88% compared to previously isolated A-Africa topotype Genotype IV strains in Egypt in 2016 and 2018. The locally used vaccine strain, which is serotype A from the Iran-05 lineage in the Asia topotype, showed substantial genetic variations, especially at key antigenic positions of the VP1 protein, with a nucleotide difference of 26.34% [70].

An examination of capsid sequences from serotype A revealed unequal evolution among different topotypes, with Africa and Asia evolving more quickly than Euro–SA. The observed high level of genetic variation supports the notion of an elevated mutation rate, along with the effects of purifying and positive selection processes that target or are located near the antigenic sites [71].

In Ethiopia, sequence analysis of the VP1 for serotypes A and O highlighted exchanges of amino acid residues at positions 28 and 42 to 48, 138, 141, 142, 148, 156, 173, and 197 in serotype A compared to the vaccine strain (ETH/6/2000). Likewise, serotype O exhibited amino acid mutations at positions 45, 48, 138, 139, 140, 141, and 197 when compared to the vaccine strain (ETH/38/2005). These amino acid residue changes were found in the B-C loop, G-H loop, and the C-terminus of the VP1 protein at sites 1 and 3 in both serotypes.

Antigenic matching of serotypes A and O serotypes using a one-dimensional virus neutralization test showed up to 76.47% similarity with the vaccine strain, although some field strains demonstrated r-values below 0.3 [72]. Additionally, a study in the United Arab Emirates (UAE), a study analyzed 2021 outbreaks by comparing virus samples with six different vaccine strains. It found that the vaccines used in Abu Dhabi were effective against the circulating virus strains, particularly after administering a booster dose. This led to over 80% of sheep and goats developing strong immunity.

This study also underscored the significance of booster doses, indicating that there were marked improvements in immunity following a second dose. These findings support the UAE’s vaccination strategy, suggesting that high coverage and consistent booster doses, combined with biosecurity measures, are crucial for effective control of FMD [73].

Monoclonal neutralization antibodies play a crucial role in defending against FMD and serve as valuable tools for the detailed analysis of epitope structures. Analyzing the structures of FMDV-AAF-R55 and FMDV-AWH-R55 at near-atomic resolution clarified how the R55 molecule interacts with specific regions of the VP2 (βB and BC/HI-loops) and VP3 (B-B knob) proteins of FMDV, which are essential for neutralizing the virus. The VP3E70 residue (BC-loop) is identified as a significant factor contributing to the differences in neutralization between these two FMDV strains [74]. Additionally, the epitope recognized by R50, which facilitates cross-serotype neutralization, is highly conserved in the O/A serotypes of FMDV. These findings suggest a need for the development of broadly cross-protective vaccines [75]. Therefore, further investigations into different serotypes are recommended to evaluate the immunogenic relationship between newly identified and existing vaccinal strains, with the goal of achieving optimal protection against existing viruses.

## Antigenic sites substitutions

Focusing on key mutations and structural changes provides valuable insights into the complex dynamics of viral genetics, host interactions, and vaccine development. Serotype Asia-1 was mutated with an Asp-to-Asn substitution at position 72 of the VP2 B-C loop (rD72N). This substitution reduced the virus’s reactivity with monoclonal antibody (mAb) 1B4 and decreased its virulence in suckling mice, highlighting the importance of the VP2 protein in understanding structure–function relationships and in the development of FMDV vaccines [76].

Additionally, serotype A demonstrated two amino acid substitutions: threonine → isoleucine (T48I) at the B-C loop and alanine → valine (A143V) at the G-H loop. These alterations distorted the VP1 G-H loop in a study involving cattle and pigs in Bangladesh [77]. Furthermore, deletions of the PK (pseudoknots) of the 5’UTR were found to affect the host range of serotype O FMDV [78].

Moreover, through a combination of scanning proline mutagenesis and random mutagenesis, several mutations were identified, including I22P, T100P, V124P, and L127P. These findings provide important insights into how proline disruptions in the B2 β-strand of the 3C^pro^ can enhance expression levels [79] (Table 1).

**Table 1:** **Antigenic sites substitutions of FMDV**

Serotype	Mutation	Significance	Species	References
Asia-1	Substitution mutation; Aspartic acid → Asparagine (rD72N) at B-C loop of VP2 protein	Hindered the virus reactivity with monoclonal antibody (mAb) 1B4 and lessened its virulence	Suckling mice	[76]
A	Substitution mutation; threonine → isoleucine (T48I) at B-C and alanine → valine (A143V) at G-H loops of VP1 protein	Alteration in the structure of the G-H loop of the VP1 protein can lead to a mismatch in the vaccine	Cattle and pigs in Bangladesh	[77]
O	Deletion mutation of the pseudoknots (PK) region of the 5’ Untranslated Region (UTR)	Changing the host range	O/Cathay topotype in pigs O/SEA topotype in cattle	[78]
FMDV 3C^pro^	Proline mutagenesis such as I22P, T100P, V124P, and L127P	Enhancing expression levels of the 3C^pro^ through disruptions in the B2 β-strand	In vitro using baby hamster kidney (BHK-21) cells	[79]
FMDV 3D^pol^	Point mutations; Tryptophan → phenylalanine (W237F) Tryptophan → isoleucine (W237I)	(W237F) → (W237F^HF^) (W237I) → (W237I^LF^)	In mice	[84]
A/SKR/Yeoncheon/2017 vaccine strain (A-1)	Point mutations; Glutamic acid → Lysine (E82K) of VP2 protein	Promising vaccine candidate against FMDV serotype A in the Asian region	In mice	[85]
Indian serotype A virus vaccine	Deletion mutations at residues 87 to 144 in the C-terminal region of the 3A protein	Eliciting an immune response	In guinea pigs	[86]

Viruses that possess a single nucleotide polymorphism (SNP) in the Arginine-Glycine-Aspartic acid (RGD) motifs of VP1 can regain virulence if the RGD pattern is restored. Furthermore, vaccines that do not contain RGD can perform just as well, or even better than binary ethyleneimine (BEI) inactivated vaccines in terms of providing protection and stimulating immune responses.

A study investigating alanine substitutions through RGD peptides found that the leucines located at the first and fourth sites following RGD (RGD + 1 and RGD + 4 sites) are significant for inhibiting the virus’s ability to bind and infect via the αvβ6 or αvβ8 receptors. However, these leucines were not deemed critical for preventing virus binding to αvβ3, which is considered a poor receptor for infection by serotype O [80].

Also, when the RGD motifs are replaced with RSD, RDD, SGD, REG, GGG, or RGG, the infection of FMDV can still occur [81]. Additionally, research has demonstrated that FMDV can still replicate in cell culture despite lacking a portion of the G-H loop (142–154 aa) by utilizing heparan sulfate proteoglycans (HSPG) as an alternative receptor. This highlights the significance of HSPG binding for vaccine viruses and suggests that targeting this pathway could be beneficial for FMDV vaccines [82].

In a study conducted on goats in Korea*,* a vaccine was developed utilizing antigens extracted through heparin affinity chromatography. This method resulted in the production of nAbs that target homologous viruses while successfully avoiding the elicitation of antibodies against NSPs. This indicates that this approach could yield a higher-quality vaccine compared to using polyethylene glycol (PEG), particularly in terms of the purity of the FMD vaccine [83].

Manipulating the fidelity of RNA-dependent RNA polymerase (RdRp), known as 3D^pol^, could help in developing safer and more stable vaccine candidates for FMDV. Research has focused on targeting Trp237, a specific amino acid in RdRp, to create point mutations that alter replication fidelity. This research, conducted on mice, found that replacing Trp237 with phenylalanine increased fidelity (W237F^HF^), while substitutions with isoleucine and leucine decreased fidelity (W237I^LF^) [84].

One promising experimental vaccine strain, A/SKR/Yeoncheon/2017 (A-1), contains a single amino acid mutation, E82K, of the VP2 protein. This vaccine candidate has demonstrated effectiveness by protecting mice against the A/MAY/97 virus, which has a serological mismatch with the vaccine strain [85]. This is considered a promising vaccine candidate for protection against FMDV serotype A within Asia.

Additionally, researchers developed a recombinant deletion mutant virus vaccine targeting the 3A protein of the Indian serotype A. This vaccine lacks amino acid residues 87 to 144 in the C-terminal region and has proven effective in eliciting an immune response in guinea pigs. It also displayed similar antigenic characteristics to the parental strain [86].

Understanding key mutations and structural changes in FMDV proteins is crucial for the development of safe and reliable vaccine candidates. The use of recombinant deletion mutant virus vaccines can also help differentiate between infected and vaccinated animals with greater precision.

A recent meta-analysis indicated that vaccine protection in pigs is comparable to that observed in other animal models such as mice, guinea pigs, and suckling mice. The findings showed no significant differences in protection levels with pigs exhibiting mean hazard ratios of 0.56, 0.67, and 1.70 compared to mice, guinea pigs, and suckling mice, respectively. This suggests that these animal models could serve as effective substitutes for pigs when evaluating the efficacy of FMDV vaccines [87].

## A comparison of traditional and recent trends in FMD vaccines

### General consideration of FMDV vaccination

Current FMD vaccines have several limitations, which include:

- the need for multiple vaccinations due to inadequate long-term protection;
- a dependence on a continuous cold chain because of thermal instability;
- the risk of vaccinated animals becoming asymptomatic carriers if exposed to the virus;
- the presence of NSP traces in some vaccines from certain manufacturers, complicating the Differentiating Infected from Vaccinated Animals (DIVA) strategy;
- increased production costs to address the instability of vaccines; and
- the relatively high cost of vaccines, which poses a particular challenge for small-scale farmers [20].

FMD vaccines in endemic regions are typically multivalent and must have a potency of at least three protective doses (PD50) per dose. These vaccines are inactivated with adjuvants such as saponin-hydrogel or oil-adjuvant. It is recommended that animals be revaccinated every 4 to 6 months to eradicate disease spread; however, some manufacturers propose a more frequent vaccination schedule of five times per year in the African context.

Vaccination campaigns should be conducted regularly, considering epidemiological situations, the value of the animals, and the economic circumstances. It is also crucial to ensure an appropriate interval between vaccinations to avoid leaving the animals vulnerable during the “window of susceptibility” when their immune system may be inadequately protected against infectious diseases [2].

**Figure 3:**
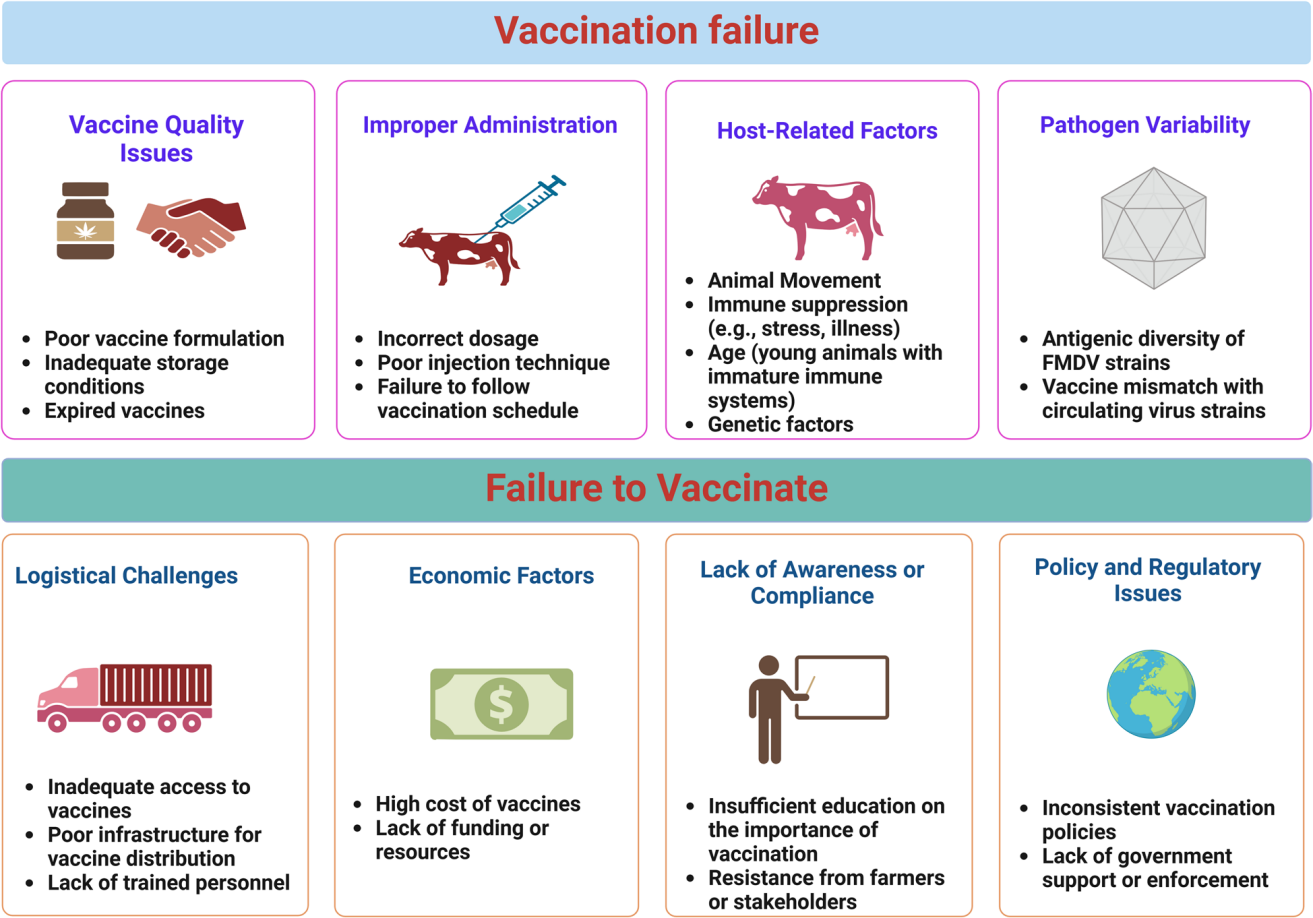
**Graphic representation of the potential factors contributing to FMDV vaccine ineffectiveness.**

Vaccination failure presents a complex challenge that arises from multiple factors. These factors can be categorized into four main areas: 1. Vaccine quality issues: problems such as poor formulation, inadequate storage conditions, and the use of expired vaccines can significantly contribute to vaccination failure [88]. 2. Improper administration: incorrect dosage, poor injection technique, and failure to adhere to the vaccination schedule are critical factors that can affect the effectiveness of vaccines. 3. Host-related factors: factors related to the recipient, such as animal movement, immune suppression, age of the animals, and genetic background influence how well the vaccine works. 4. Pathogen variability: differences among pathogens especially the antigenic diversity of FMDV strains and a mismatch between the vaccine and circulating virus strains, can lead to ineffective vaccination [67] (Figure 3).

However, failure to vaccinate can also be attributed to broader systemic issues beyond the direct factors associated with the vaccine and its administration.

Logistical challenges, such as inadequate access to vaccines, poor infrastructure for distribution, and lack of trained personnel can hinder vaccination efforts. Economic factors, including the high cost of vaccines and insufficient funding or resources, further exacerbate this issue [89]. Additionally, a lack of awareness or compliance including stemming from inadequate education about the importance of vaccination and resistance from farmers or other stakeholders—can also impede vaccination efforts. Policy and regulatory issues, such as inconsistent vaccination policies and a lack of government support or enforcement, make the situation worse.

Addressing these systemic barriers is essential for improving vaccination coverage and enhancing the effectiveness of FMDV control measures [90].

**Figure 4:**
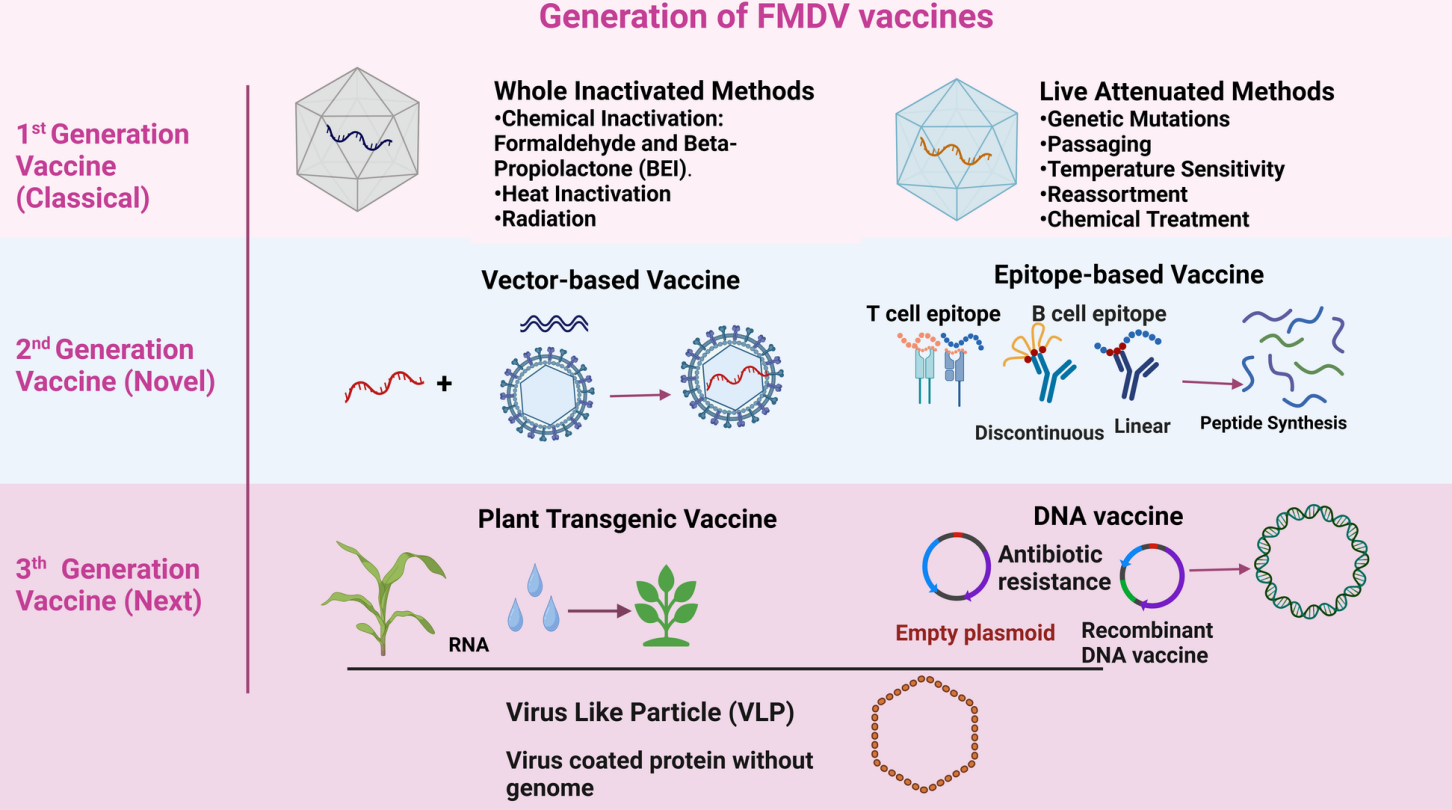
**Classification of foot-and-mouth disease virus (FMDV) vaccines by generation, showcasing the progression from traditional methods to advanced biotechnological approaches.** The first generation (Classical) vaccine includes whole inactivated vaccines, which use chemical agents such as formaldehyde and beta-propiolactone or heat inactivation to eliminate viral infectivity, and live attenuated vaccines which are developed through genetic mutations, passaging, temperature sensitivity, reassortment, or chemical treatment to create a weakened virus that still elicits an immune response. The second generation (novel) vaccine introduces vector-based vaccines, utilizing viral vectors to deliver FMDV antigens effectively, and epitope-based vaccines, which employ synthesized peptides that mimic specific T-cell and B-cell epitopes to stimulate targeted immune responses. In the third generation (next generation) vaccine, further advancements are seen with plant transgenic vaccines, where transgenic plants are engineered to express FMDV antigens, DNA vaccines that use recombinant plasmids for gene delivery, and virus-like particles (VLPs), which are virus shells devoid of genetic material designed to mimic the virus structure and trigger a strong immune response. These generational advancements reflect improvements in vaccine safety, efficacy, and production efficiency, addressing the need for effective FMDV prevention strategies in livestock populations.

Traditional vaccine production methods have typically relied on the accumulation of mutations around antigenic and binding sites, influenced by the dynamics of quasi-species swarms and positive selection. Therefore, integrating computational biology with advancements in our understanding of viral immunology and pathogenesis is essential for developing safe and effective vaccines that provide long-lasting immunity. This includes features such as single-dose vaccination, enhanced capsid stability, proper antigenic matching, and DIVA capability [19]. The various types of vaccines used for FMD are illustrated in Figure 4.

### Inactivated vaccines

Current marketable FMD vaccines are primarily composed of inactivated vaccines treated with BEI to remove NSPs. They are available in monovalent, bivalent, or multivalent formulations, using various bases such as oil-emulsion, aqueous solutions, or aluminum. These vaccines are characterized by their high antigen concentration and can be effectively preserved in liquid nitrogen, primarily serving as emergency vaccines in regions free from FMD.

These highly concentrated vaccines typically provide protection against challenges within one week of administration. The vaccination schedule requires primary doses spaced one month apart, with subsequent booster doses administered every 4–6 months for animals as young as two years old, followed by additional annual injections [91].

Despite their effectiveness, these vaccinations have several limitations. There is a risk of viral escape during manufacturing, a limited shelf life, and the need for booster injections every 4–12 months. Conventional inactivated vaccines also face further challenges related to safety, hygiene, ease of administration, cost, and the maintenance of persistent immunity against various serotypes [20].

While mineral oils are commonly used, they often do not provide a strong and lasting immune response. A study investigated this issue by examining various adjuvants: Quil-A, *E. coli* DNA, and Montanide™ ISA 206 in relation to antibody responses in sheep. The results indicated that Quil-A alone produced the highest levels of antibodies, followed by the combination of Quil-A and *E. coli* DNA. Additionally, *E. coli* DNA outperformed Montanide™ ISA 206 on certain days. These affordable alternatives could enhance the effectiveness of FMD vaccines for mass vaccinations of sheep [92].

Nanoparticles and nanomaterials are now recognized as next-generation adjuvants due to their beneficial physicochemical properties and reduced side effects. Among these materials, clay nanomaterials such as layered double hydroxide (LDH) and hectorite, a hydrotalcite-like clay, have shown the ability to improve immune responses and act as effective adjuvants [93]. They serve as an efficient vaccine carrier, leading to increased antibody levels, the maturing of dendritic cells, and the enhancement of strong specific T-cell immune responses. LDH-based vaccinations also promote robust cytotoxic T-lymphocyte responses and significantly inhibit tumor proliferation [94].

As a result, LDH is a promising vaccine adjuvant due to its small particle size, stable dispersion, large specific surface area, positive charge, significant cargo capacity, sustained release, easy absorption, low toxicity, cost-effectiveness, and enhanced cellular immune response. LDHs generate antibodies persistently, indicating a slow release capability, and could potentially serve as effective adjuvants for FMDV [95].

One study investigated the potential adjuvanticity of LDH nanoparticles by outlining a method for synthesizing and characterizing them, as well as adsorbing a model antigen, bovine serum albumin (BSA). The results showed that LDHs were not intrinsically harmful and elicited a superior humoral response in mice compared to free BSA and Alum-BSA. The 56 nm LDH particles were identified as the most effective carriers, producing a more balanced Th1/Th2 response [96].

To address the limitations of inactivated vaccines, various organizations have modified them using a modified viral pathogen. These modified inactivated vaccines give DIVA compatibility without the need to isolate NSPs. Additionally, the virus can be cultivated safely for candidate animals, reducing the requirement for level III biosafety in vaccine production [19].

For example, in the vaccinal strain IND/Asia-1/491/1997, a part of the 3A protein and the entire 3B1 and 3B2 proteins were removed. In the cell culture system, the mutant virus exhibited infectivity titers equivalent to those of the wild-type virus [97]. Furthermore, a study successfully produced and evaluated antigens from the O PA-2 and A22 IRQ FMD viruses using a 100 L bioreactor. The antigens demonstrated effective protection in pigs, indicating their potential for large-scale vaccine production in South Korea [98]. Additionally, this inactivated FMD vaccine, when combined with a chemokine CCL20 plasmid as an adjuvant, stimulates both cellular and humoral immunity in mice, underscoring its potential as a comprehensive solution [99].

Thus, improving FMD vaccine production methods and developing modified engineered vaccines, such as inactivated vaccines that incorporate intrinsic DIVA NS markers into specific proteins, offer enhanced protection with fewer drawbacks.

Using genetic engineering can significantly enhance effectiveness of vaccines against FMDV. A study describes the development of a full-length cDNA clone of the FMDV vaccine strain with a genetically modified version that includes specific amino acid changes in 1, 3, and 4 antigenic sites. These modified viruses grew similarly to the original O/HN/CHA/93 virus. When swine were vaccinated with an inactivated vaccine derived from this strain, they achieved full protection against certain FMDV topotypes and partial protection against others. Remarkably, swine receiving the genetically modified vaccine were completely protected against all tested topotypes [100].

Another study developed a candidate vaccine targeting the seven serotypes of FMDV by replacing a specific gene from a known vaccine strain with genes from all seven different serotypes. This approach successfully created viruses representing each type. When pigs were vaccinated with a trial vaccine that included inactivated versions of the modified viruses from the O, A, and Asia1 serotypes, they exhibited strong protection against the disease [101].

Researchers also employed a plasmid-based reverse genetics technique to create a new strain of FMDV. By replacing a gene in the standard vaccine strain O/CHA/99 with one from the epidemic strain A/WH/CHA/09, they produced a chimeric virus that displayed growth similar to the original strain. Following successful testing in Baby Hamster Kidney (BHK) cells, this virus was selected as a potential vaccine candidate. When cattle were vaccinated with the inactivated version of this chimeric virus, most developed a robust immune response within a week [102].

A chimeric vaccine was designed by combining proteins from various topotypes: the VP4, VP2, and VP3 proteins from the ME-SA topotype (O Manisa) with the VP1 protein from either the SEA topotype (O/SKR/Jincheon/2014) or another ME-SA topotype (O/PAK/44). The chimeric viruses were inactivated, and when used for immunization, they provided full protection to mice against all three topotypes. Among pigs, the PA2-VP1 vaccine elicited higher neutralizing antibody levels early in the vaccination process compared to the JC-VP1 vaccine. While both vaccines were effective, PA2-VP1 offered slightly better protection against the SEA, ME-SA, and Cathay topotypes than JC-VP1[103].

In China, researchers aimed to develop a vaccine that delivers broad protection against all serotype O FMDVs and addresses potential cross-protection against the PanAsia-2 strain. They created two chimeric FMDV vaccines, rHN/TURVP1 and rHN/NXVP1, designed to protect against all FMDV serotype O lineages and the PanAsia-2 strain. Both vaccines were inactivated, and when administered, the rHN/TURVP1 vaccine demonstrated better protection and higher antibody levels in pigs and cows than rHN/NXVP1 and effectively targeted the Cathay lineage [104].

South Korea developed the Asia1/MOG/05-R vaccine strain to combat FMD outbreaks, specifically targeting the Asia1 group-V genotype. This vaccine demonstrated strong antigen productivity, effective viral inactivation, and commercial viability. Pigs vaccinated with Asia1/MOG/05-R developed high virus neutralization titers, providing robust protection against FMD. This strain is a valuable candidate for inclusion in commercial antigen banks [105].

In conclusion, genetic engineering has significantly improved FMDV vaccines. By combining inactivated and genetically modified strains, broader protection against various serotypes can be achieved. These advancements enhance vaccine effectiveness and strengthen our strategies for controlling the disease.

### Live attenuated vaccines (LAVs)

Researchers have engineered attenuated viruses through both conventional methods such as cell culture passage, and novel techniques including gene mutations, alterations in replication fidelity, and codon deoptimization. These strategies have resulted in varying degrees of protective efficacy in animals. The goal is to strike a balance between safety through attenuation and maintaining sufficient immunogenicity for effectiveness. One of the primary advantages of LAVs is their ability to induce long-lasting immunity [50].

Experimental modifications to the L^Pro^ of FMDV have been investigated, although animal studies are still limited. Deleting the leader protease gene makes the virus non-virulent in swine and cattle, while an in-frame shift mutation in that gene leads to an attenuation in cattle. Neither the leaderless (LL) nor the in-frame vaccinations generate viral infection or clinical symptoms following aerosol inhalation. However, the leaderless variant spreads less effectively than the in-frame variant and may gradually regain pathogenicity [106].

Additionally, a study examined two substitution initiation sites, comparing AUG in both the Lab and Lb sites, with AUG of Lb being preferred. Researchers mutated the AUG of Lb to non-AUG codons (AUC, AAA, CUG, and UGG). While these mutations did not stop viral viability, they did affect replication differently in each cell type. The mutants grew well in BHK-21 cells but exhibited delayed growth in pig kidney (PK-15) cells. This delay was linked to reduced cleavage of eIF4GI and impaired interferon suppression caused by the L^Pro^ protein [32].

Innovative approaches to attenuate FMDV include removing the conserved SAF-A/B, Acinus, and Protein Inhibitor of Activated STAT (PIAS) domain (SAP) from L^pro^. This method has led to swine being protected within two days after vaccination against a similar challenge [107]. However, challenges remain, particularly the risk of reversion to virulence associated with certain attenuation methods, such as codon pair deoptimization. This highlights the necessity for careful consideration and ongoing evaluation of these vaccine strategies [108].

Synonymous codon deoptimization was applied to the conserved P2 and P3 coding regions, allowing for the development of LAV for various FMDV serotypes. By integrating these modified regions into the A24Cruze infectious clone, different levels of attenuation were observed in cell cultures, mice, and swine [109].

Overall, these innovative methods for attenuating FMDV have shown promising results in enhancing the vaccine's protective efficacy against related strains, demonstrating the potential for novel techniques to improve animal health and vaccine effectiveness.

### Transgenic vaccines in plants

Plant-derived vaccines offer a convenient and cost-effective technology, as plant cells can function as bioreactors capable of producing complex antigens while maintaining their antigenic properties. Transgenic vaccines in plants are increasingly recognized as a promising approach for targeting linear epitopes. Notably, using transgenic plants to produce edible vaccines for FMDV addresses challenges associated with inactivated vaccines, such as transportation and cold storage requirements.

Successful expression of the VP1 structural protein has been achieved in *Arabidopsis thaliana*, *alfalfa*, and potato plants [110]. However, edible vaccines face challenges related to the detection and expression of transgenic proteins in plants. The incorporation of the β-glucuronidase gene (GUS) as a reporter has been shown to facilitate quick identification and screening of transgenic plants, leading to robust humoral immunity in laboratory hosts [111].

Furthermore, effective vaccination in mice has been observed in Argentina and China with the expression of the FMDV VP1 protein in transgenic plants [112]. An oral administration protocol for the FMD vaccine has been successfully established using VP1 from serotypes Asia-1 and O in maize, with stable transmission of the transgenes to the next generation [113]. Recombinant vaccines produced in transgenic plants, such as cereals, provide advantages including reducing concerns related to viral or prion contamination and lowering transportation and storage costs [114].

Additionally, Tobacco Necrosis Virus A has been utilized to construct chimeric virus particles (CVPs) that produce strong immune reactions against FMDV when inoculated in mice [115]. Transgenic chloroplasts of green algae are also being explored as a source of mucosal vaccines [116]. Overall, the effectiveness of transgenic plants in expressing FMDV antigens and inducing strong immune responses underscores the potential of plant-based vaccines to overcome the challenges faced by conventional FMD vaccines.

### DNA vaccines

DNA vaccination typically involves a piece of a plasmid that caries the specified microorganism gene, which is controlled by a promoter that induces gene expression. The plasmid is transported to the endoplasmic reticulum, where it is cleaved by cellular proteases. This process leads to the subsequent presentation of peptides on MHC I molecules at the cell surface, ultimately eliciting an immune response. This plasmid can encode components such as a modified full or small-length FMDV genome, an empty capsid, either alone or conjugated with regulatory genes that have a role in immunity.

Notable characteristics of DNA vaccines include their ability to stimulate both T and B cells, compatibility with the immune system, their safety due to the absence of infectious agents, their ease of manufacturing, and their stability without the need for cold chain storage. They also allow for the incorporation of DIVA-capable marker genes, and can be adapted to include field strain sequences, enabling multiple antigenic sites. However, challenges exist, such as the requirement for multiple doses with substantial DNA quantities to achieve efficacy, as well as potential variability in immune response [117].

Vaccines encoding the FMDV capsid protein and the 3D^Pol^ protein have demonstrated protective immunity in pigs. In mice, a bifunctional DNA vaccine generated antisense RNA targeting the FMDV 5' UTR and successfully induced immune responses [118]. Additionally, another study developed a new DNA vaccine that fused FMDV B and T-cell epitopes with a variable fragment of the 1F12 mouse monoclonal antibody (mAbs), which recognizes Class-II Swine Leukocyte antigens. During viral challenge, half of the DNA- immunized pigs were completely protected, correlating with the induction of specific IFN-γ-secreting T-cells and the rapid development of nAbs [119].

In an investigation assessing four DNA vaccine candidates against FMDV in cattle, researchers produced VLPs and mature FMDV P1 cleavage products. These formulations effectively mimicked certain features of the FMDV structure and triggered the production of specific viral protein fragments (cleavage products), which are important indicators of the vaccine's potential efficacy [120]. Furthermore, incorporating cytokines as effective adjuvants in DNA vaccines has shown promise, with interleukins playing crucial roles in enhancing immune responses [121]. However, environmental factors such as chronic heat stress can negatively impact the immune response to FMD DNA vaccination, adversely affecting cellular immune responses [122].

Nucleic acid aptamers are short strands of DNA or RNA that bind tightly and specifically to particular molecular targets. One study used Capillary Electrophoresis SELEX (CE-SELEX) to create DNA aptamers that target the 3ABC protein for diagnosing FMD. The binding strength is commonly assessed using a value known as the dissociation constant (Kd), which represents the equilibrium constant for the reaction where the aptamer and its target separate from one another. The study identified an aptamer named FMDV1, which exhibited a strong binding affinity for the 3ABC protein, with a Kd value of 22.69 ± 1.79 nM. Importantly, FMDV1 demonstrated significantly higher specificity for the 3ABC protein compared to a control bovine serum albumin (BSA) [123].

Additionally, the Gibson assembly (GA) method is a widely used technique for DNA construction due to its simplicity and cost-effectiveness. This method allows for the construction of a plasmid from overlapping DNA fragments in a single step, eliminating the need for separate subcloning procedures. During the GA reaction, exonucleases trim nucleotides from the ends of DNA fragments, creating single-stranded overhangs that facilitate the annealing of the DNA. A study conducted by Semkum et al. employed the GA method to combine viral coding regions with a vector (pKLS3) in a single reaction. The resulting infectious clones could be utilized for genetic manipulation and research, ultimately supporting the development of customized FMD vaccines. This approach represents a novel method for creating full-length infectious FMDV cDNA clones, which can enhance vaccine development efforts [124].

DNA vaccines have significant potential to address FMD. They offer several advantages over traditional vaccines and have shown success in animal models. Continued research and exploration of DNA vaccine components could lead to more effective and scalable solutions for preventing and managing FMD.

### Epitope-based vaccines

Epitope-based peptide vaccines represent an innovative approach that utilizes small protein fragments, or epitopes, derived from pathogens to trigger immune responses. The recent advancements in immunoinformatics and vaccinomics have propelled vaccine science into a new era, significantly improving the development of safe, potent, and innovative next-generation vaccines.

Protein subunit vaccines face challenges in inducing cross-responses because conformational or discontinuous epitopes differ from linear epitopes. Discontinuous epitopes arise when distant amino acids within a protein's primary sequence converge in the protein’s folded structure. Mapping these discontinuous epitopes using synthetic peptides or escape mutants can be quite challenging [125].

Peptide vaccines hold several advantages over inactivated vaccines, including cost-effective production, stability, the absence of infectious components, and DIVA. These vaccines depend on peptide-conjugated carrier proteins, such as bacterial toxoid or ovalbumin. These carriers must adhere to strict standards for effectiveness, safety, and cost-effectiveness [19].

However, peptide vaccines also have some drawbacks such as partial immunity, which is primarily due to their limited coverage of antigenic sites. This limitation is especially evident with discontinuous antigenic sites on VP1. Additionally, the quasi-species behavior of FMDV can lead to outbreaks in vaccinated animals due to the presence of different antigenic variants [67].

To address these challenges, mannan-decorated inulin acetate microparticles (M-IA MPs) have been designed to serve as carriers and adjuvants for administering the recombinant FMD multi-epitope subunit vaccine containing 5 B cell and one T-cell epitopes, referred to as M5BT. The M5BT-loaded M-IA MPs were characterized by their morphology, size, and release kinetics, which were determined using field emission scanning electron microscopy, dynamic light-scattering spectrophotometry, and spectrophotometry [126].

The M5BT-loaded M-IA MPs showed elevated levels of antigen-specific IgG, IgG1, IgG2a, and anti-FMDV antibodies compared to M5BT-loaded IA MPs and Freund’s adjuvant used as a control. Despite these encouraging results, there are still few reports of achieving complete immunity in pigs following vaccination with an FMD peptide vaccine [127].

#### Targeting B-cell epitopes for broad-spectrum protection

Crystallographic investigations have provided valuable insights into the configuration of the FMDV capsid, highlighting exposed epitopes on its surface that are associated with structural loops. Monoclonal antibody studies have identified the presence of five neutralizing antigenic sites (1–5). Among these sites, 1, 3, and 5 are located within VP1, site 2 encompasses residues from VP2, and site 4 incorporates a portion of VP3. Notably, site 1 is linear and sensitive to trypsin, while the other sites depend on conformational configurations [128].

The βG-βH loop (residues 134–158) and the C-terminus (residues 200–213) of VP1 play crucial roles in forming site 1, featuring these crucial residues 144, 148, 154, and 208. Residues at positions 70–73, 75, 77, 131, and 188 of VP2 make up site 2, with reports indicating that residues at positions 79 and 134 influence the binding of site 2 mAbs [129]. The βB-βC loop of VP1 contains residues 43 and 44, which contribute to site 3, while residues at positions 56 and 58 of VP3 are deemed critical for site 4 [130]. Site 5 is defined by an amino acid at position 149 of the VP1 G-H loop [131].

Recent efforts have focused on predicting epitopes based on sequencing data of the FMDV capsid and its three-dimensional (3-D) structure. These predicted epitopes then undergo mutational studies using reverse genetics. A novel epitope (VP2 191) has been found to be part of antigenic site 2 in serotypes O and A using a similar technique [132]. Additionally, residue 220 of VP3 of serotype A [133] and residues 116 and 195 around the threefold axis of VP3 of serotype O have been denoted to be of antigenic significance [134]. On the other hand, residues 58–71 of VP3 of serotype O, previously identified as an antigenic site, were thought to have a key role in the antigenicity of the SAT2 strain [22] (Table 2).

**Table 2:** **The known antigenic sites of FMDV serotypes O, A and SAT2**

	Protein	B-cell epitopes		T-Cell Epitopes	
		Serotypes O and A	Serotype SAT2	Serotypes O and A	Serotype SAT2
Structural Protein	VP1	- G-H loop (134–158) and C- C-terminal (200–213) residues (Site 1) [129, 190]^**a**^ - 144, 148, 154, and 208 positions with significant importance - Β-C loop (43 and 44) residues (Site 3) [130]^**a**^ - G-H loop at 149 residues (Site 5) [131]^a^	- 48–50, 84–86, 109–111, 137–140, 157–160, 169–171, and 199–201 [191]^**a**^ - 140–150 [22]^**a,c**^ - 156 [192]^**c**^	31‐45 and 46‐60 [193]^**a**^	- 21–40, 161-C terminal [194]^**a**^ - 135–144, 150–160 [195]^**a,bc**^ - 210 [191]^**a**^
	VP2	- 70–73, 75, 77, 131 and 188 position (Site 2) [129, 190]^**a**^ - 79 and 134 influence the binding of site 2 mAbs - 191 residues in serotypes O and A [132]^**ab**^	- 71–72, 133–134 [191]^**a**^ - 89–105 [125]^**a**^	54–72 [135]^**a**^	49–68, 113–132, 179–198 [22]^**ac**^
	VP3	- 56 and 58 residues. (Site 4) [132]^**ab**^ - 116 and 195 residues (Serotype O) [134]^**a**^ - 220 residues (Serotype A) [132]^**ab**^	55–88, 176–186, 208 [22]^**ac**^	116‐130 [193]^**a**^	130–134 [195]^**abc**^
	VP4	30–41 [196]^a^		20–34 [197]^a^ 71‐85 [193]^a^	
Non-Structural Protein	3A	410, 412 and 417 [198]^a^		**21–35** [127]^b^	
	3D	16–30 [198]^a^		56–70 [199]^b^	

^**a**^ in vitro

^**b**^ in vivo

^**c**^ in silico

##### Targeting T-cell epitopes for broad-spectrum protection

All essential bioinformatics processes include the prediction and discovery of antigenic B and T cell epitopes, as well as the assessment of immunity titers [38]. Despite the significant sequence variability of the VP1 protein across different FMDV serotypes, the detection of T-cell epitopes in more conserved domains has been driven by the VP1 G-H loop [22]. As a result, T-cell epitopes within the VP4 (20–34) protein, which is structurally highly conserved, have been identified for both cattle [135] and swine [136]. This peptide has shown a strong binding affinity to four distinct MHC Bovine Leukocyte Antigen (BoLA) types, utilizing MHC class II DQ (Delta-Quantitative) molecules that play a crucial role in antigen presentation to immune cells.

T-cell epitopes have been identified in NSPs, showing limited sequence diversity among different serotypes. T-cell epitopes in pigs were identified in 3ABC using overlapping peptides covering all NSPs of FMDV. Notably, peptide 3A (21–35), when delivered linearly with the VP1 G-H loop peptide, induced lymphoproliferation and helped to elicit nAbs. These peptides are promising candidates for development into novel, fully protective peptide vaccines [127].

Additionally, the inclusion of peptide, 3D (56–70) resulted in a significant and lasting production of IFN-γ for up to three months post-vaccination, along with high levels of nAb titers. Cross-presentation of these peptides can activate the CTL response to exogenous vaccination antigens. By targeting T-cell epitopes to DCs, which can prime this response, and incorporating toll-like receptors (TLR), this promising approach for eliciting systemic CTL responses in pigs is being explored [137].

Consequently, CTL epitopes rely on in silico predictions and in vitro validation using various laboratory animal models. For example, Gao et al. identified numerous nine-residue peptides from VP1 that triggered CTL responses in mice and provided immune protection in guinea pigs [138]. Several peptides have also been predicted to bind BoLA-Al and Swine Leukocyte Antigen (SLA) MHCI alleles [139]. Furthermore, Ning et al. analyzed the relationship between an FMDV CTL epitope and SLA-2 *04:02:02 using X-ray diffraction, revealing the 3-D configuration of the peptide binding site and identifying critical residues involved [140].

In conclusion, Table 2 lists known B and T cell epitopes found in structural and non-structural proteins of serotypes A, O, and SAT2.

##### From antigenic sites to synthetic peptide vaccines

After analyzing FMDV’s antigenic sites, the next step involves synthesizing these sites into artificial peptides that could serve as potential vaccine candidates. This process is complex because most antibodies recognize conformational, non-contiguous epitopes [38]. Therefore, when designing synthetic peptide replicas for these non-contiguous (scattered) locations, it is crucial to consider the spatial arrangement of the relevant parts of the epitope so that they are positioned close together, mimicking the native structure. This arrangement enhances the interactions between epitope and paratope. Additionally, peptides in solution are more flexible than folded proteins, which require strategies such as cyclization—especially for well-defined structural motifs like loops—to effectively reduce their mobility [141].

Most antigenic sites on viral proteins are discontinuous; however, some sites are continuous and can be effectively replicated using linear peptide simulations. Linear peptides that mimic the VP1 G–H loop from different serotypes of FMDV elicited nAbs in mice and guinea pigs, while showing a limited immune response in pigs [142]. Subsequent studies introduced chimeric peptides representing linear epitopes such as VP1 G–H loop and C-terminus, which successfully induced nAbs and provided modest protection in pigs [143] and cattle [144]. Another linear construct, the ACT peptide, comprised the G-H loop, C-terminus, and residues (21–40) of VP1 for sites A, C, and T, respectively, found in cattle [145].

However, the effort to replicate immunogenicity through the simplified approach of peptide vaccines faces significant challenges including: (1) the uncertainty surrounding neutralization reactions and immunity generated from animals vaccinated by peptide vaccines; (2) the diversity in the antigenicity of FMDV; (3) limited knowledge of many immunogenic peptides recognized by T- cells and MHC molecules; and (4) the susceptibility of short peptides to proteolysis [146].

The NSP 3ABC proteins generated during FMDV infection are absent in commercial inactivated vaccines. Consequently, animals infected with FMDV produce antibodies against these non-structural proteins, whereas vaccinated animals do not. This distinction is critical for the DIVA strategy, which facilitates effective disease monitoring and control.

In a study, three mAbs (2G5, 9E2, and 1E10) were used to target NSP 3ABC. The mAb 2G5 identified the linear B cell epitope “92EYIEKA97”, while mAb 9E2 recognized “23EGPYAGPLE31” and mAb 1E10 identified the epitope “209EPHH212”. The key amino acids in these epitopes were confirmed through alanine-scanning mutagenesis analysis. Notably, the mAb 2G5 epitope is located in the 3A region, which is absent in certain natural mutants that affect the virus's tropism. The 3B1 and 3B2 were targeted by mAb 9E2, while 3B3 did not react, indicating its preservation across various FMDV isolates. Furthermore, preliminary evaluations using confirmed positive and negative sera suggest that mAb 9E2 could serve as a useful diagnostic tool for the DIVA approach [147].

A new recombinant protein was developed using a baculovirus system. This protein combines the G-H loop epitope of the FMDV VP1 with the vesicular stomatitis virus. Glycoprotein was generated and subsequently validated through ELISA using a mAb that targets FMDV. After two vaccinations were administered a month apart to pigs, a significant increase in antibodies was observed compared to a glutathione S-transferase carrier that contained the same epitope, as well as in comparison to commercially available vaccines [148].

Another innovative trial used a Taiwan-Jincheon (TWN-JC) construct, where the Jincheon epitope (JC) was positioned at residues 152 and 153 of the Taiwan-R (TWN-R) VP1. This construct was tested for its ability to protect mice against challenges from three distinct topotype viruses [149].

Additionally, in Iran, researchers developed epitope recombinant vaccines based on the FMDV serotype O VP1 protein, which were coupled with Fc Immunoglobulin (FcIgG) and IL-2. This vaccine generated a heightened immune response in BALB/c mice and is currently being evaluated as a potential adjuvant for epitope recombinant vaccines targeting the VP1 protein [150].

Furthermore, two recombinant proteins, AVM and OVM, which included antigenic sites from different topotypes of serotypes A and O, were successfully designed and produced. These proteins elicited strong immune responses in both mice and pigs and provided protection against multiple strains of FMDV [151].

Designing a vaccine that includes complex mixtures of peptides representing various antigenic variants has shown increased immunogenicity compared to using individual peptides alone. A recombinant vaccine featuring multiple tandem-repeat epitopes demonstrated strong immunity against the (O/China/99) field strain in pigs, leading to a significant increase in anti-FMDV-specific antibody levels 30 days after vaccination. This vaccine incorporated three copies of the peptides corresponding to amino acids 141 to 160 and 200 to 213, arranged in triplicate with GGSSGG as a linker sequence for epitope separation [152].

Additionally, a recombinant trivalent vaccine targeting three different strains of FMDV was tested in pigs with at least 25 μg of antigen. This vaccine successfully generated FMDV serotype O-specific and neutralizing antibodies through a primary-booster regimen, providing complete protection against all three virus strains. Importantly, none of the fully protected pigs produced anti-3ABC antibodies, which indicates sterilizing immunity. The vaccine also induced long-lasting high antibody levels, offering effective protection for up to six months, making it a promising candidate for the future prevention and control of FMD [153].

Mice injected with a multi-epitope chimeric recombinant protein designated “5BT”, which contains five consecutive repetitions of a B-cell epitope (VP1 amino acid residues 136–162) originating from diverse FMDV strains and one T-cell epitope (3A residues 21–35), developed detectable levels of antibodies [154]. Another chimeric FMD vaccine strain was specifically developed and tested for the Asia region. The strain combines antigenic epitopes from FMD virus serotype A, including components from sea-97/G1 (VP4, VP2, VP3) and either A22 Iraq (VP1) or G-VII (VP1). Mice vaccinated with these strains were protected when exposed to three different type A viruses (Sea-97/G1, Sea-97/G2, G-VII clade) seven days after vaccination. Similarly, pigs vaccinated with one of the candidate vaccines were also protected when challenged with the same viruses 28 days post-vaccination [155].

The three fusion proteins—FT (Flagellin-Truncated VP1), FTME (Flagellin-Truncated VP1-Multiple Epitopes), and FME (Flagellin-Multiple Epitopes)—were tested as new vaccine candidates for FMDV in guinea pigs, demonstrating efficient immune defense against infection [156]. Additionally, three synthetic peptide vaccines, based on immunogenic epitopes from the FMDV strain (A/HuBWH/CHA/2009) were evaluated in both guinea pigs and cattle. All of these vaccines induced nAbs, with the PB peptide showing the highest immunogenicity. The peptide provided significant immune protection, achieving 100% protection in guinea pigs after two doses and 60% protection in cattle after a single dose [157].

A novel multi-epitope vaccine, HAO, derived from serotypes A and O, was combined with the recombinant protein heparin-binding hemagglutinin (HBHA), which acts as a potent TLR4 agonist. This fusion protein, known as HAO-HBHA, stimulated robust specific cellular and humoral immunity, both in vitro and in vivo. The findings suggest a new strategy for developing a secure and effective multi-epitope bivalent vaccine candidate for FMDV types A and O, as well as broadening the use of TLR agonist-based vaccine systems [158].

In China, a successful vaccine against serotype O FMDV was created by engineering two chimeric viruses, rHN/TURVP1 and rHN/NXVP1, that contained substituted VP1 genes. These chimeric viruses closely resembled the wild-type virus in terms of biological characteristics. Pigs and cows vaccinated with rHN/TURVP1 showed higher antibody titers against structural proteins compared to those vaccinated with rHN/NXVP1, providing cross-protection against various FMDV lineages [104].

Online prediction software was used to identify recombinant epitopes (VP1 [141–160]-GS-VP1 [23–42]-GS-3A [21–35]-GS) from various proteins of Iranian serotypes O, A, and Asia-1. This recombinant protein has been proposed as a universal vaccine candidate for controlling FMD in Iran [159].

Additionally, a recombinant polypeptide vaccine produced from B-cell epitopes of VP1 and VP4, along with T-cell epitopes from the 2C and 3D proteins, was previously developed using a phytoviral expression in *Nicotiana benthamiana* plants or *Escherichia coli* (*E. coli*). This vaccine demonstrated effective protection against FMDV serotype (O/Taiwan/99) in guinea pigs [160].

Moreover, an immunoinformatic approach identified two T-cell epitopes within the VP1 (VQRSRQSTL) and VP3 (YHAEWDTGL) proteins that exhibited strong binding affinity with MHCI alleles and showed good conservation with SAT2 African serotypes. Additionally, one B-cell epitope in the VP3 protein was predicted, suggesting the potential for vaccine development [161].

In summary, developing effective vaccines for FMDV using synthetic peptide replication necessitates detailed measures to simulate the native structure. Nonetheless, recent advancements in recombinant proteins and multi-epitope vaccines have shown promising results in various animal models, highlighting their potential for controlling and eliminating this disease.

##### FMDV dendrimer peptide vaccines

The dendrimer peptide vaccine produces a strong FMDV-specific immune response in pigs, resulting in high levels of nAbs and T-cell activation. Dendrimer peptides contain multiple copies of a B-cell epitope (VP1 136 to 154) linked to a T-cell epitope (3A 21 to 35). This design induces potent humoral and cellular responses within a single molecule, overcoming the limitations of individual peptides and providing protection against viral challenges.

The multivalency of the B-cell epitope in dendrimers (B2T and B4T) demonstrates greater effectiveness in inducing nAbs and IFN γ release compared to linear peptide juxtaposition in mice. The bivalent B2T architecture can elicit B and T cell responses that are comparable to or even superior to those generated by the tetravalent B4T. The presence and arrangement of T-cell epitopes are crucial for the immunogenicity of linear monovalent peptides, offering valuable insights for designing more effective FMD subunit vaccines [162].

**Figure 5:**
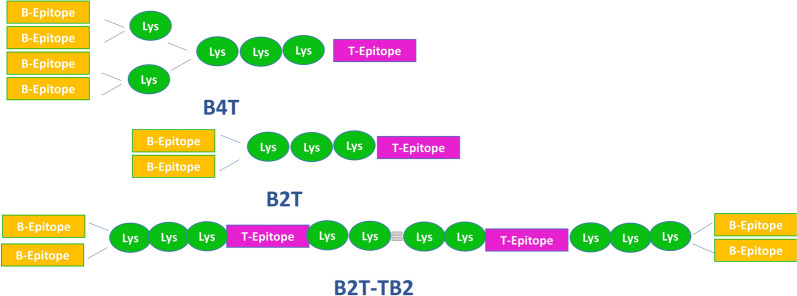
**Different models of multiple-antigen peptide (MAP) systems**. MAPs are dendritic polymers with lysine cores (represented as green circles) and multiple antigenic epitopes. The B4T model shows a lysine core with four B-cell epitopes connected to one T-cell epitope. The B2T model displays a simpler structure with two B-cell epitopes linked to one T-cell epitope. The B2T-TB2 model features a linear arrangement with two B-cell epitopes on each end, connected through lysine cores to central T-cell epitopes. The figure is based on [189] with modifications.

In similar tests conducted on cattle, both B4T and B2T peptides produced the same T-cell responses, with elevated levels of IgG1 detected in the serum and mucosa [163]. Additionally, the new dendrimer peptide B2T-TB2 triggered high nAb titers in both mice and swine [164] (Figure 5). Synthetic peptide vaccines utilizing non-structural 3D proteins of FMDV have also shown potential, with B2T-3D inducing similar levels of nAbs and greater IFN-γ production compared to B2T-3A in swine [165].

Overall, dendrimer peptide vaccines demonstrate a robust immune response at both humoral and cellular levels, with the arrangement of B-cell and T-cell epitopes being critical for generating strong immunity. The promising results observed across multiple animal models provide important perspectives for the development of future vaccines.

### FMDV live vector vaccines

Recombinant viral vectors are a type of genetic vaccine that are integrated into a modified viral genome under the control of eukaryotic promoters. In this context, genetic material is introduced into the cells of the immunized animal through infection with the recombinant virus. Viral vectors are considered more effective than DNA vaccines because their unique receptors facilitate the rapid integration of foreign genes into host cells [166]. However, the effectiveness of vaccination may be reduced if the immune system develops a response against the viral vector. Several research groups have utilized mammalian viral vectors, such as poxviruses, herpes viruses [167], *Salmonella* [168]**,** and adenoviruses, to deliver FMDV sequences that express structural proteins in vaccinated animals, thereby eliciting an immune response [169] (Table 3).

**Table 3:** **Vector-based FMDV vaccines**

Vector-based FMDV vaccine	Significance	Immunization model	References
Herpes simplex virus (HSV-1) amplicon vector	Significant reduction of viremia with partial protection	Mice	[167]
*Salmonella* vector system (KST0666) expressed FMDV VP1 gene	Induction of humoral and cellular immunity High FMDV neutralization titer and high levels of IgA, IgG Th1, Th2, and Th17 cells	Mice	[168]
Recombinant canine adenovirus type 2 (CAV2) expressed the P1 precursor protein along with the 3C protein (P1/3C) or expressed the VP1 protein of the FMDV	Strong immune response Vaccination marker	Guinea pigs	[169]
PEGylated bovine IFN lambda 3 delivered via a replication-defective adenovirus (Ad) vector	It remained detectable for over 10 days, offering antiviral protection for four days In co-administration, it enhanced the immune response, highlighting its potential as a rapid-acting therapy during FMD outbreaks	Cattle	[171]
Recombinant adenovirus-5 vectored FMD (Ad5- FMD) expressed FMDV capsid and viral 3C ^pro^ proteins	Induction of immune response with high neutralization titer in monovalent form	Cattle	[170]

Human adenoviruses and vaccinia viruses have been widely used as vectors in various studies. Up to now, the recombinant-replication defective human adenovirus has shown promise as a means to deliver the FMDV capsid protein into animals [170]. One study investigated a fast-acting treatment for FMD using PEGylated bovine IFN lambda 3, which was delivered via a replication-defective adenovirus (Ad) vector. This approach aimed to extend the protective effects of the treatment.

When administered to cattle, either alone or alongside the Ad-based FMD vaccine (Adt-O1M), the modified IFN effectively prevented FMD symptoms when given three to five days prior to viral exposure. It was detectable for over ten days, providing antiviral protection for four days. Additionally, co-administration with the vaccine enhanced the immune response, highlighting its potential as a rapid-acting therapy during FMD outbreaks [171].

Furthermore, refinements of the vaccine has focused on integrating 2B NSP, improving the synthesis of FMDV capsid proteins, and incorporating additional RGD motifs to enhance adenovirus transduction in immune dendritic cells. Although the effectiveness of recombinant viral vectors may be diminished by the development of immunity against the viral vector, they remain a promising and viable method for delivering FMDV sequences to animals and eliciting immune responses.

### Virus-like particles

Virus-like particles (VLPs), also known as empty viral capsids, are spontaneously produced in vitro and do not contain any nucleic acid. They offer numerous advantages, including an increased capacity for DIVA, reduced reliance on biosafety level III conditions, and economic benefits. Research has shown that VLPs stimulate DCs similarly to inactivated vaccines while also promoting humoral immunity [172].

VLP vaccinations are typically designed in one of two ways. They can be produced in various cultures, including bacteria, plants, mammalian cells, or insect cells, and then administered as candidate vaccines. Traditionally, VLPs are generated using a baculovirus expression system followed by purification [173]. Recent advancements in VLP production have involved the use of dual promoter vectors, such as 3C^pro^, or a bicistronic complementary DNA cassette that includes two ORFs encoding P1-2A and 3C^pro^, separated by an IRES [174].

A trivalent VLP vaccine has been developed to target senecavirus A (SVA) and FMDV serotypes O and A. The results demonstrated that this combined vaccine elicits strong antigen-specific antibody responses, comparable to those produced by individual vaccines. The immune sera effectively neutralized both FMDV and SVA at similar titers to the monovalent vaccines, indicating a strong immunogenic compatibility among the VLP components. Notably, the combined vaccine provided protection against both single and mixed infections in pigs, whereas the individual vaccines only offered protected against their respective viruses [175].

Additionally, chimeric rabbit hemorrhagic disease virus (RHDV)-VLPs can serve as an antigen carrier, triggering specific T-cell responses in pigs and increasing the number of IFN-γ-secreting cells that target the 3A epitope of FMDV and RHDV-VLP [176] (Table 4). Recent research has highlighted the effectiveness of prokaryotic-expressed VLPs in protecting cattle, with *E. coli* being the most common expression system for FMDV VLPs [177].

**Table 4:** **Virus-like particles FMDV vaccine**

Virus-like particle (VLP) FMDV vaccine	Significance	Immunization model	References
VLP contains an SNP at 93 and 98 positions of the VP2 of FMDV serotype O (IND/R2/75)	Enhanced stability at 37 °C over 75 days Induction immune response	Guinea pigs	[180]
A trivalent VLP vaccine targeting senecavirus A (SVA) and FMDV serotypes O and A	Strong antigen-specific antibody responses of both FMDV and SVA are comparable to the responses from individual vaccines	Pigs	[175]
Chimeric VLP containing the 3A protein	Robust immune response	Pigs	[176]
Assembled recombinant FMDV VLPs from *E. coli* co-expression system	High protection is achieved with a potency of 11.75 PD50 per dose, where the bovine protective dose (PD50) reaches 50%	Cattle	[177]
VLP used hepatitis B virus core (HBVc) particles combined with FMDV epitopes	High titer of humoral and cellular immunity	Mice	[178]
RHDV VLP contains FMDV epitopes	Trigger strong neutralizing immune response	Mice and Pigs	[200]
Assemble HEV-FMDV into VLP and expressed in *E. coli*	Thermostable and revealed ideal antigenicity and immunogenicity properties	Swine	[181]
chimeric VLP-based rabies	Exposure of major antigenic sites of FMDV in three distinct regions	In vitro	[179]
3C ^pro^ and P1 polyprotein of FMDV serotype O were expressed in the yeast *Hansenula polymorpha*	Elevated levels of INF-γ and CD4 + T cells	Mice	[182]
Antisense RNA with VLPs that displayed the epitope peptide 141–160	Triggered an immune response Protection was observed in 40% of suckling mice and 85% of guinea pigs against FMDV	Mice and guinea pigs	[183]
*Saccharomyces cerevisiae* was used as an expression system to boost the production of the VP3-VP1 construct combination (20aaVP3-VP1-Co1)	Strong VP1-specific IgG and IgA immune responses	Mice	[184]

Furthermore, chimeric VLPs that display B-cell epitopes derived from serotypes A and O FMDV, along with a T-cell epitope using a truncated hepatitis B virus core (HBc) carrier, have been constructed. These VLPs induced specific IgG and nAbs against serotypes A and O in mice, significantly stimulating the production of Th1-type cytokines while suppressing Th2 cytokine production [178].

Finally, chimeric VLPs based on the rabies virus were created to present a significant antigenic site of FMDV. This approach highlighted the identification of suitable regions within the rabies glycoprotein (RVG) that preserved correct protein folding and was externally exposed on the surface, allowing them to recognized by anti-FMDV antibodies in expressing cells and on the surface of VLPs [179].

Recent studies have shown significant advancements in the thermostability of vaccines. For example, a mutation in the VP2 region has enhanced the thermostability of VLPs, leading to substantial protection in guinea pigs [180]. Additionally, a bioinformatics approach was used to design a HEV-FMDV chimeric vaccine candidate. This candidate demonstrated high over-expression in *E. coli* as a soluble protein and exhibited the ability to self-assemble into VLPs. Overall, this vaccine candidate displayed excellent thermostability, optimal antigenicity, and strong immunogenicity. These properties contribute to reducing zoonotic HEV transmission to humans and preventing highly contagious FMD in swine [181].

In a study, the 3C ^pro^ and P1 polyprotein of FMDV serotype O were expressed in the yeast *Hansenula polymorpha* to produce self-assembling VLPs. The effectiveness of these recombinant VLPs as a potential FMDV vaccine was assessed. Mice immunized with these VLPs exhibited elevated levels of INF-γ and CD4 + T cells [182].

Another study developed a new vaccine for FMDV by integrating antisense RNA with VLPs that displayed the epitope peptide 141–160. This vaccine was tested in mice and guinea pigs, producing an immune response that protected 40% of suckling mice and 85% of guinea pigs from FMDV. These results suggest that the epitope-RNA VLP vaccine holds promise for future FMDV vaccine development [183].

Additionally, *Saccharomyces cerevisiae* was used as an expression system to enhance the production of VP1 of FMDV. By creating fusion proteins, researchers discovered that the combination of VP3-VP1—particularly when 20 amino acids from the N-terminal of VP3 were added to VP1 construct 1 (Co 1) peptide (N-SFHQLPARSPLP-C) (known as 20aaVP3-VP1-Co1)—resulted in significantly higher expression levels. Further enhancement was achieved by lowering the culture temperature, thereby matching the expression levels of other proteins typically produced in yeast. When this yeast-expressed fusion protein was orally administered to mice, it triggered strong VP1-specific IgG and IgA immune responses, indicating its potential for developing an oral FMDV vaccine [184].

These developments underscore the importance of rigorously reviewing and evaluating VLP-based vaccine strategies to ensure that future advancements effectively contribute to controlling FMDV while addressing emerging challenges in vaccine design and distribution.

Bacteriophages, which are naturally occurring, share structural similarities with viruses and offer significant benefits in the prevention and treatment of viral diseases. They serve as effective vaccine delivery systems due to their various advantages. Bacteriophages have adjuvant properties that enhance immune responses, making them efficient for protein and epitope immunization. Additionally, they are considered safe for both humans and animals, which is a substantial advantage in vaccine production [185].

The genomes of bacteriophages are easily modifiable, allowing for the integration of antigens or the creation of DNA vaccines. They can also be produced on a large scale, which helps reduce production costs. Furthermore, they are resilient in various environments, facilitating easier transportation and storage [186].

In one study, the T7 bacteriophage capsid was used to express the VP1 protein of the FMDV AKT-III strain, evaluating its potential as an innovative vaccine. The AKT-T7 strain was developed effectively and exhibited no cytotoxicity toward BHK-21, sheep kidney cells, or MDBK cells. It was phagocytosed by murine macrophages and successfully induced FMDV antibodies in BALB/c mice. The AKT-T7 prompted the generation of FMDV antibodies, leveraging the benefits of both the phage and the FMDV, positioning it as a promising candidate for an FMD vaccine [187].

Another study developed a chimeric nanoparticles (CNPs) vaccine that presents the VP1 (131–160) of FMDV serotype O on the surface of MS2 phage. The recombinant protein can autonomously aggregate into CNPs with a diameter of 25–30 nm in vitro. Mice were inoculated with CNPs, tandem repeat peptide epitopes (TRE), and commercially available synthetic peptide vaccines (PepVac). The results indicated that CNPs elicited elevated specific antibody levels and improved the cellular immune response, suggesting potential for future management of FMDV [188].

## Conclusion

FMD is a highly contagious and economically devastating viral disease that necessitates urgent and effective control measures. This review highlights the significant challenges in combating FMD, which arise from the complex immune evasion strategies employed by the FMDV. A detailed understanding of FMDV proteins and their roles in inhibiting host immune responses—such as disrupting interferon production, degrading cellular factors, and inducing apoptosis—is essential for developing targeted countermeasures.

This review also emphasizes the critical importance of effective FMD vaccination, especially considering the diversity of FMDV serotypes and the lack of cross-protection among them. Progress in utilizing immunoinformatics to design epitope-based vaccines shows promise in addressing this challenge by identifying conserved and immunogenic epitopes that can elicit broad protection.

Additionally, the review underscores the need for continuous monitoring and alignment of vaccine strains with circulating FMDV strains to ensure optimal protection against the evolving virus. Looking ahead, promising areas for future research and development include the exploration of novel vaccine technologies, such as the use of nanoparticles, adjuvants, and combination vaccine strategies, to enhance the efficacy, stability, and safety of FMD vaccines. Furthermore, addressing systemic barriers to vaccination—such as logistical challenges, economic factors, and policy-related issues—will be crucial for improving vaccination coverage and overall efforts to control FMD.

## Data Availability

Not applicable.
